# Cross-sectional study to assess etiology and associated factors for anaemia during first trimester of pregnancy in Anuradhapura District, Sri Lanka: a protocol

**DOI:** 10.12688/f1000research.28226.3

**Published:** 2022-04-12

**Authors:** Gayani Amarasinghe, Vasana Mendis, Thilini Agampodi, Suneth Agampodi

**Affiliations:** 1 Faculty of Medicine and Allied Sciences, Rajarata University of Sri Lanka, Anuradhapura, north cetral, 50000, Sri Lanka

**Keywords:** Anemia, Pregnancy, Sri Lanka, Anuradhapura

## Abstract

**Background:** Anaemia in pregnancy, which can lead to adverse maternal and fetal outcomes, is a significant global health problem. Despite Sri Lanka’s strong public health system and commitment towards prevention, maternal anaemia remains a major problem in the country. While prevention is focused on iron deficiency, detailed etiological studies on this topic are scarce. Moreover, estimates of socio demographic and economic factors associated with anaemia in pregnancy, which can provide important clues for anaemia control, are also lacking. This study aims to evaluate the hemoglobin distribution, and geographical distribution, contribution of known aetiologies and associated factors for anaemia in pregnant women in Anuradhapura, Sri Lanka.

**Methods: **This is a cross sectional study of pregnant women in their first trimester registered for antenatal care from July to September 2019 in Anuradhapura district. The minimal sample size was calculated to be 1866. Initial data collection has already been carried out in special field clinics for pregnant women between June to October 2019. An interviewer-administered questionnaire, a self-completed dietary questionnaire and an examination checklist were used for data collection. In addition, all participants underwent complete blood count testing. Further investigations are being conducted for predicting the etiology of anaemia based on a developed algorithm (such as high-performance liquid chromatography [HPLC] and peripheral blood film analysis).

**Discussion:** Being the largest study on anaemia during pregnancy in a single geographical area in Sri Lanka, this study will provide important clues about geographical clustering of anaemia cases with similar etiology, associated factors and etiologies which would help to develop interventions to improve the health of pregnant women in the area. The possibility of selection bias is a potential limitation associated with the study design.

## Introduction

Anaemia in pregnancy is associated with many adverse outcomes for the mother (such as loss of productivity, heart failure and even death) as well as her offspring (such as low birth weight, anaemia and developmental problems) ^1– 4^. Despite exceptional maternal care standards and numerous nutritional interventions, anaemia in pregnancy remains a major problem in Sri Lanka ^5^. Assuming iron deficiency is the major underlying cause of anaemia ^6^, anaemia screening during preconception and antenatal clinics (at their initial appointment and 28 weeks), universal iron supplementation, provision of double dose iron (elemental iron 120 mg/day) for anaemic mothers, nutritional education, prophylaxis for malaria, and treatment for worm infestations have long been incorporated to Sri Lanka’s pregnancy care package ^7^. Interventions with a lifecycle approach such as iron supplementation at schools are also in place ^8^. Despite all these measures, maternal anaemia remains a major public health problem in certain districts of the country ^9– 21^. 

In 2018, 22.8% of pregnant women in the Anuradhapura district were identified as anemic during their initial visit to an antenatal clinic and despite routine interventions for iron deficiency anaemia, the prevalence was almost triple among the second trimester women ^22^; amounting to a severe public health problem ^23^. This raises the question of whether etiologies other than iron deficiency are significantly responsible for anaemia in this population. Previous reports on the presence of hemoglobinopathies, heterogeneous nutrient deficiencies and hereditary disorders among Sri Lankans supports this assumption ^24– 26^. A resent national wide study also indicates that iron deficiency may contribute only to a 34% of anaemia in pregnancy ^20^.

Identifying dietary, socio economic ^11, 17^, demographic ^12, 14, 16, 20^, biological and behavioral factors that lead to anaemia in pregnancy will shed light on this unsolved problem as published literature exploring these factors is scarce. Even though a significant variation in prevalence between districts has been reported ^14, 16, 20^ intra-district (micro-geographical) variations have not yet been studied thoroughly.

This study aims to estimate the haemoglobin distribution and the prevalence of anaemia, and identify factors associated with anaemia among first trimester pregnant women in Anuradhapura district, Sri Lanka. It will also explore the contribution of several etiological causes to anaemia in this population.

## Methods

### Study setting

**Figure 1.**
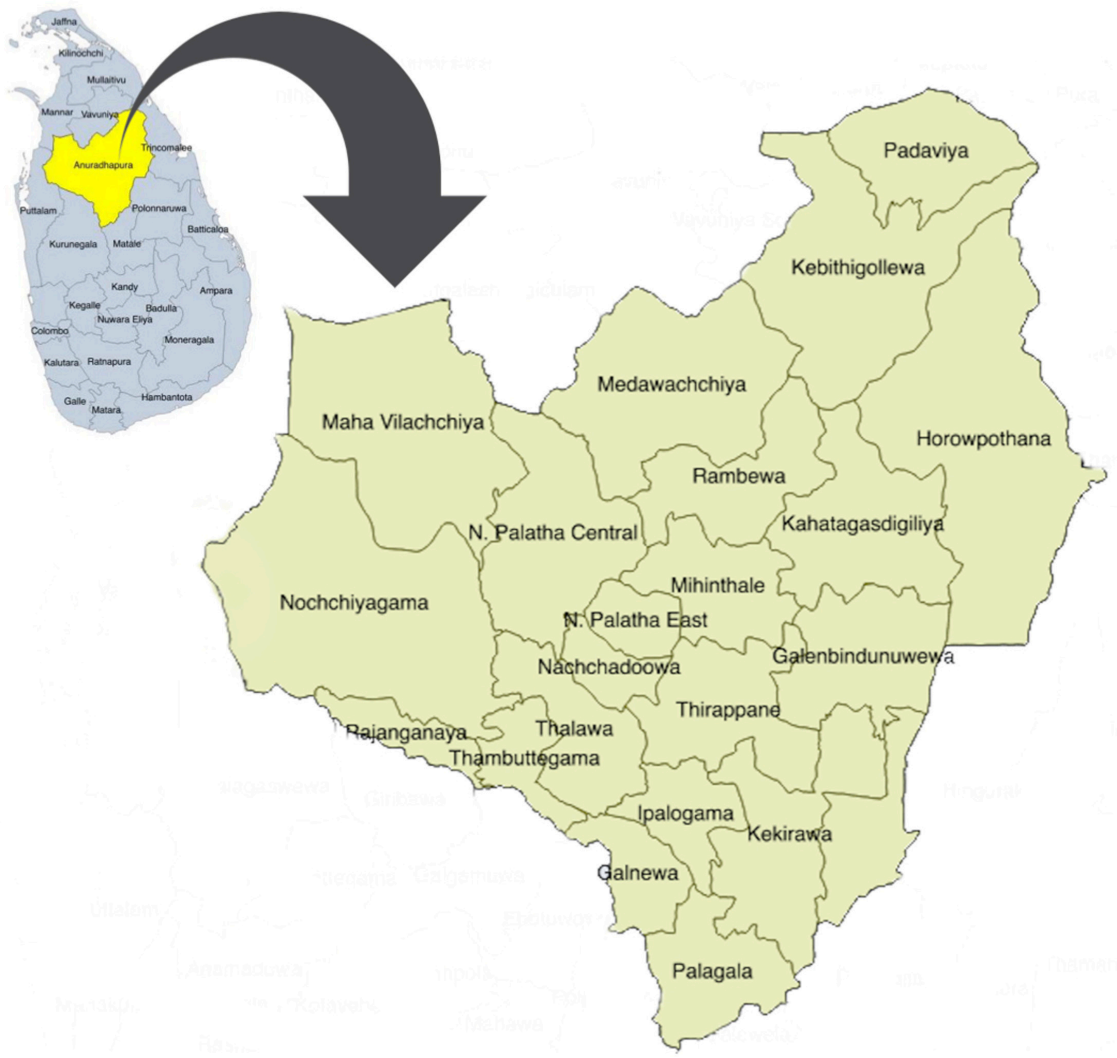
Map of the Anuradhapura district, Sri Lanka. This map depicts the position of Anuradhapura district (yellow) in the map of Sri Lanka (blue) and the area is enlarged to depict the 22 medical officer of health areas in the district. The figure has been reproduced with permission from Agampodi TC, Wickramasinghe ND, Prasanna RIR, Irangani MKL, Banda JMS, Jayathilake PMB, *et al.* The Rajarata Pregnancy Cohort (RaPCo): study protocol. BMC Pregnancy Childbirth [Internet]. 2020 Jun 26 [cited 2020 Nov 30];20(1):374. Available from: https://bmcpregnancychildbirth.biomedcentral.com/articles/10.1186/s12884-020-03056-x.

This study was carried out in Anuradhapura, the largest of 25 districts in Sri Lanka. Anuradhapura district is divided into 275 public health midwife (PHM) divisions within 22 medical officers of health (MOH) areas ( Figure 1) which conduct village level antenatal clinics. Early registration for antenatal care and clinic attendance is very high (96%) in this district ^5^.

### Study design

This cross sectional study was conducted as the baseline assessment of the Rajarata Pregnancy Cohort (RaPCo) ^27^. This study includes the baseline assessment of etiology of anaemia in early pregnancy, supported by an evidence-based algorithm adopted and tested in a sub sample (n=200) of women selected consecutively from the same study sample. The study includes two components; the basic investigations conducted at the first special antenatal clinic held in early pregnancy (component 1) and a second assessment if further investigations were needed to identify the etiology of anaemia (component 2). Although the cohort of women were followed up until delivery, this particular study protocol is focused on identification of etiology of anaemia of first trimester pregnant women.

### Eligibility criteria

All pregnant women in their first trimester (less than 13 weeks pregnant) and registered in the pregnancy care programme from July to September 2019 were invited to take part in the study. There were no exclusion criteria.

### Sample size

We hypothesized that causes of anaemia affecting at least 10% of anemic pregnant women would be significant from a public health point of view. Therefore, the minimum sample size needed to detect causes in at least 10 % of the anemic pregnant women was calculated using the following formula ^28^:

$$n={\left(z_{1-\frac{\alpha }{2}}/d\right)}^{2}p(1-p)$$

$$\text{n}=[{\left(1.96/0.03\right)}^{2}*.1*.9]=383$$

where n = minimum sample size, z = level of confidence 95% – 1.96, d = precision - 3%, p = expected proportion of participants with a selected cause for anaemia in the population – 10%.

When a 10 % nonresponse and loss to follow-up rate was applied for the minimum sample size needed for the second component the required sample size was determined as 425. Anaemia prevalence of first trimester pregnant women in the Anuradhapura district in 2018 was 22.8% ^29^. Therefore, the number of participants needed for the first component in order to get 425 anemic pregnant women was 1866.

### Recruitment

An invitation to participate in the study was sent to all eligible participants through their PHM. From July to October 2019, a special clinic was conducted fortnightly at each MOH area where the eligible participants were recruited consecutively. The special clinic was continued till October 2019 so that eligible participants registering with the PHM towards the end of August could also join.

### Data collection: Component 1

Baseline data collection was carried out by four teams, each led by a fully qualified MBBS doctor. Each team included several medical graduates, a nursing officer and four to six trained data collectors. All team members received three days of training and all procedures were carried out according to a manual.

Three study instruments were used; an interviewer administered questionnaire on socio demographic, economic, behavioral and medical factors (filled in by data collectors), a self-report questionnaire on diet (filled in by the participants at home and returned through the public health midwives), and a form to record findings of clinical examination conducted by a medical graduate ^30^. All the tools were developed specifically for this study. They were validated by a panel of multidisciplinary experts including a consultant community physician, social epidemiologist, hematologist and physicians and public health midwives (PHMs) working in the area where study is conducted. All tools were pretested among 20 pregnant women with the same eligibility criteria but registered in the maternal care program prior to the establishment of the cohort. The questions were comprehendible according to pretest results with the consensus of the expert panel.

The short clinical interview was conducted to identify presence or absence of symptoms of anaemia (shortness of breath, fatigue on exertion or at rest, faintness and chest pain). Anthropometric measures (height, weight, waist circumference and hip circumference), pallor and murmurs were recorded after a clinical examination. The dietary assessment included a food frequency questionnaire, assessment of consumption frequency of selected iron rich food items and the type of usual main meal

***Collection of blood samples.*** Blood sample collection, handling, storage and analysis were conducted according to a protocol ^30^. For all the participants, a complete blood count was performed at the time of baseline data collection during the special clinic. A slide was prepared for peripheral blood film analysis for each participant at the time of blood collection by trained laboratory technicians.

### Development and testing of the algorithm

**Figure 2.**
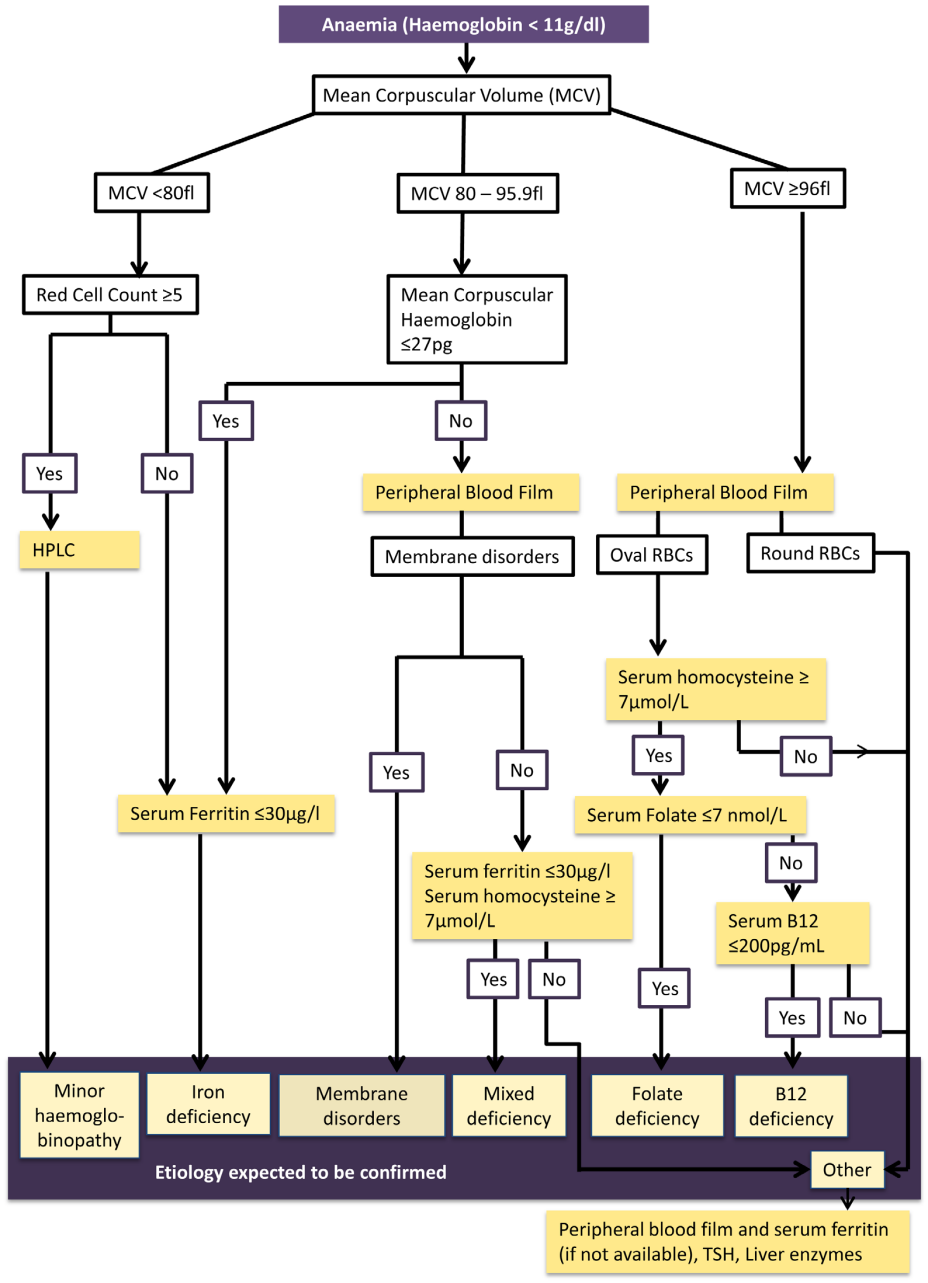
Algorithm for investigating etiology of anaemia among first trimester pregnant women in Anuradhapura district. This figure shows the evidence-based algorithm developed to guide the laboratory investigations to determine etiology of anaemia among first trimester pregnant women in the Anuradhapura district. Anaemia is determined by haemoglobin level less than 11 g/dl. Anaemia will be further classified as mild (10 – 10.9 g/dl) moderate (7–9.9 g/dl) and severe (<7 g/dl) based on the WHO classification. Mean corpuscular volume is used to identify microcytic (MCV less than 80 fl), normocytic (MCV 80 to 95.9 fl) and macrocytic anaemia (MCV more than 96). Those who have microcytic anaemia with high red cell counts (five or more*10 ^6^/µl) will be tested for minor haemoglobinopathies using HPLC testing. Those who have microcytic anaemia with normal red cell counts or have normocytic anaemia with mean corpuscular haemoglobin (MCH) 27 pg or less will undergo serum ferritin testing to determine iron deficiency. A peripheral blood film of participants with normocytic anaemia with MCH more than 27 will be examined to see if there are membrane disorders. Then serum Homocysteine and ferritin will be estimated in them to determine mixed deficiency. If this etiology is not confirmed with these investigations, thyroid stimulating hormone (TSH) and liver enzymes will be tested along with ferritin assessment. A peripheral blood film will be examined in macrocytic anaemic participants. If macrocytes are round, serum Homocysteine will be estimated. If it is raised, serum folate and B12 levels will be conducted to determine which nutrient is deficient. If oval macrocytes are present, TSH, liver enzymes and serum ferritin will be assessed.

A working algorithm was developed using available research evidence ^31– 38^ and hematological expert opinion to identify the etiology of anaemia ( Figure 2). The algorithm was developed as performing each investigation on all anemic women was not technically and financially feasible. Considering the feasibility, the different pathways of the algorithm were tested in 200 consecutively taken blood samples representing the district in the on going cohort. Serum ferritin level was assessed to determine iron status in all 200 of this sub-sample in addition to analysis of peripheral blood film. In anaemic pregnant women of this sample, further investigations (HPLC, serum B12, Folate levels and serum homocysteine levels) were conducted to test the algorithm. 

According to the algorithm high red cell count (RCC ≥ 5*10 ^6^) is used to differentiate minor hemoglobinopathies from iron deficiency among microcytic anemic cases (MCV <80fl) ^31, 32^. Early iron deficiency is suspected in normocytic hypochromic anaemia (MCV 80 – 95.9 fl). Suspected etiology is confirmed through serum ferritin and High Performance Liquid Chromatography (HPLC) ^33^. A peripheral blood film will be analyzed in those with normochromic normocytic anaemia and macrocytic anaemia (MCV ≥96 fl). Serum Homocysteine, serum B12 and folate levels will be used for confirming mixed deficiency, B12 deficiency and folate deficiency respectively ^34– 38^.

### Data collection: Component 2

When vitamin B12 or folate level assessment or performing HPLC was necessary according to the algorithm, participants were invited for a special hematology clinic. This clinic was conducted at the teaching hospital in Anuradhapura, the main center providing tertiary care for pregnant women in the Anuradhapura district. The invitation to participate was sent within a week following initial assessment and the appointment was booked within two weeks for serum vitamin levels assessment and according to their convenience for the thalassemia assessment. A telephone reminder was provided for all invited participants on the day prior to the scheduled appointment.

Serum ferritin, Homocysteine, B12 and Folate assessments were performed in a commercial laboratory with external quality control methods. HPLC is performed at the thalassemia unit of teaching hospital, Anuradhapura. Other investigations (complete blood count, peripheral blood film analysis and liver functions) are performed in a public health research laboratory with internal and external quality control.

### Outcomes

This study will have several important outcomes. The prevalence of anaemia in the first trimester will be assessed. This will be a valid statistic for the district as the current indicators calculated using routine data from the reproductive health management information system (RHMIS) need to be validated. Evidence on etiology of anaemia is not available for the province. This study will be valuable at the national level as large community-based studies on etiology of anaemia are scarce in Sri Lanka. Based on the outcomes the study will be able to influence maternal health policies at both national and regional level.

The study will also depict the important associations of anaemia in pregnancy, namely socio-demographic (age, ethnicity, religion, education level and economic details of the family); obstetric (period of gestation, consumption of folic acid, parity, age at each conception, outcomes and complications); medical and biological (medical conditions, regularity and length of menstrual cycle and approximate estimation of menstrual blood loss using a pictogram); contraceptive history (methods that has been used, duration of use and side effects); and pre pregnancy and inter pregnancy care (breast feeding duration, intake of iron and folate supplements during postpartum period and/or pre-pregnancy). These assessments will help to develop a bio-psychosocial model for anaemia in early pregnancy which could be applicable to other LMICs as well.

The results of the tested algorithm will guide policies and strategies for screening for anaemia which will be a benefit for future pregnant women and their families.

### Data management

Data from the interviewer administered questionnaire will be entered directly electronically. The rest of the data including laboratory investigations will be entered by single entry technique by research assistants and 10% of data will be manually verified by the investigator (GA).

All the hard copies of questionnaires and copies of investigation reports will be stored in an access restricted place, separate from the consent forms and identification details. Only investigators will have access to these copies. The database will be password protected on a separate computer. Access is limited to the investigators. 

### Data analysis

Data analysis will be performed using SPSS version 22. Mean haemoglobin distribution and anaemia prevalence will be reported with confidence intervals. Distribution of anaemia in MOH divisions will be mapped using GeoDa software. Causes for anaemia will be presented as percentages with 95% confidence levels. To determine factors associated with anaemia, chi squared tests for categorical variables (having a history of anaemia, consumption of folic acid and current breast feeding status, mothers’ ethnicity, religion, parity, education level) and t tests for continuous variables (age, severity of menstrual blood loss, BMI) will be used. For all tests, 2-sided p value with alpha ≤0.05 level of significance will be used. A logistic regression model will be attempted to predict the anaemia among first trimester pregnant women.

### Ethical considerations

Ethical approval for the ‘Rajarata Pregnancy Cohort’ of which this study is a part of, has been obtained from the ethics review committee of Faculty of Medicine and Allied Sciences, Rajarata University of Sri Lanka (ERC 2019/07).

Trained medical graduates introduced the study to participants and distributed information leaflets in native languages (Sinhala or Tamil) ^30^. Written informed consent was obtained from all participants ^30^. Participation is voluntary and refusal to take part or withdrawal from the study will not lead to any disadvantages during routine care provision. Already collected data will be retained if participation is withdrawn. Participants will receive the originals of all investigations and will be referred for further management as necessary if any abnormality is detected.

### Confidentiality

To maintain confidentiality participants will be allocated an ID number which will link their data. Consent forms will be stored separately in a locked filing cabinet at the Maternal and Child Health Research Unit (MCHRU) of our institution. All questionnaires and lab reports will be stored in one center to which only the investigators will have access.

### Dissemination of findings

Findings will be reported to the scientific community as peer reviewed research articles, abstracts and presentations. They will be conveyed to the district level stakeholders at monthly MOH conferences, special dissemination meetings and as policy briefs.

### Study status

From July to October 2019, 3018 pregnant women were recruited in this study. Baseline assessments have all been done (interviewer administered questionnaire; clinical examination; dietary assessment; complete blood count). Blood films have been prepared and serum samples have been obtained for further assessments. Confirmatory investigations for minor hemoglobinopathies such as Thalassemia is still being conducted due to difficulties in following up participants due to the coronavirus disease 2019 (COVID-19) pandemic. Peripheral blood film analysis is also yet to be completed due to lockdown related to COVID-19. Batch processing and testing of stored samples and data analysis is also ongoing.

## Discussion

This is the largest study on anaemia in pregnancy in a single district of Sri Lanka. Therefore, more specific spatial data on etiological distribution and pocketing may emerge. As studies investigating several causes of anaemia at the same time are scarce, this study will provide a wider understanding about etiology and distribution of anaemia in Sri Lankans. This will enable policy change to go beyond routine prevention methods focused on iron deficiency anaemia. Data on associated factors will also be helpful for planning further preventive interventions.

Since the participants are pregnant women who registered for routine antenatal care, a selection bias could occur. However, since the clinic registration rate is very high this bias would be minimal.

## Data availability

### Underlying data

No data are associated with this article.

### Extended data

Open Science Framework: Anaemia Tools. https://doi.org/10.17605/OSF.IO/KSX2F ^30^.

This project includes the following extended data:

1. Anaemia questionnaire - interviewer administered (in Sinhala and an English translation)
2. Diet questionnaire (in Sinhala and an English translation)
3. Examination reporting form.docx
4. Protocol for Sample Collection.docx
5. Consent form

Data are available under the terms of the Creative Commons Zero "No rights reserved" data waiver (CC0 1.0 Public domain dedication).
