# Supplementary material for: Cross-sectional study to assess etiology and associated factors for anaemia during first trimester of pregnancy in Anuradhapura District, Sri Lanka: a protocol
Source: F1000Res. 2022 Apr 12;10:223. Originally published 2021 Mar 18. [Version 3] doi: 10.12688/f1000research.28226.3 (PMC9039368; doi:10.12688/f1000research.28226.3)
Supplement: Peer review history

# Peer review: Reviewer response for version 3

**DOI:** 10.5256/f1000research.128330.r134715

**Published:** 2022-04-25

**Authors:** Richard Derman

**Affiliations:** 1 Thomas Jefferson University, Philadelphia, PA, USA

I believe the authors have responded appropriately to the questions that I had posed. 

 Recommend indexing.

Is the study design appropriate for the research question?

Yes

Is the rationale for, and objectives of, the study clearly described?

No

Are sufficient details of the methods provided to allow replication by others?

Yes

Are the datasets clearly presented in a useable and accessible format?

No

Reviewer Expertise:

Maternal child health

I confirm that I have read this submission and believe that I have an appropriate level of expertise to confirm that it is of an acceptable scientific standard.

# Peer review: Reviewer response for version 3

**DOI:** 10.5256/f1000research.128330.r134714

**Published:** 2022-04-12

**Authors:** Manjula Danansuriya

**Affiliations:** 1 Anti Malaria Campaign, Ministry of Health, Colombo, Sri Lanka

The revised version (Version 3) has addressed the issues highlighted. No further comments.

Is the study design appropriate for the research question?

Yes

Is the rationale for, and objectives of, the study clearly described?

Partly

Are sufficient details of the methods provided to allow replication by others?

Yes

Are the datasets clearly presented in a useable and accessible format?

Not applicable

Reviewer Expertise:

Community Medicine/Public Health

I confirm that I have read this submission and believe that I have an appropriate level of expertise to confirm that it is of an acceptable scientific standard.

# Peer review: Reviewer response for version 2

**DOI:** 10.5256/f1000research.56421.r125213

**Published:** 2022-03-16

**Authors:** Richard Derman

**Affiliations:** 1 Thomas Jefferson University, Philadelphia, PA, USA

This study is the largest to date to be conducted in a single region of Sri Lanka to determine the associations of socioeconomic status, dietary history, and maternal anthropometric findings with the presence of anemia that was confirmed by blood testing. Anemia is confirmed by appropriate laboratory blood analysis. 

 Survey data and nutritional information will be utilized. The published research will likely be helpful in creating information that can help inform public health policies that address proposed causation in Sri Lanka. Association of ≥ 10% will be reported.

 Diagnosing anemia during the first trimester of pregnancy is a strength of the study, as anemia in the first trimester of pregnancy is associated with the poorest maternal and perinatal outcomes. The standard use of 120 mg of oral iron in Sri Lanka when iron deficiency is diagnosed is not consistent with recent data suggesting the negative impact of hepcidin on iron absorption. 

 The study is not generalizable, being too costly to implement on a population basis. Most studies, in contrast to the data presented, suggest that iron deficiency anemia is by far the most common cause of anemia in pregnancy. Additionally, no mention of the use of intravenous iron is discussed as this therapy is becoming more widely used to address moderate to severe anemia during pregnancy. 

 It would be important to comment on the grade of anemia (mild, moderate, severe) in the findings.

Is the study design appropriate for the research question?

Yes

Is the rationale for, and objectives of, the study clearly described?

No

Are sufficient details of the methods provided to allow replication by others?

Yes

Are the datasets clearly presented in a useable and accessible format?

No

Reviewer Expertise:

Maternal child health

I confirm that I have read this submission and believe that I have an appropriate level of expertise to confirm that it is of an acceptable scientific standard, however I have significant reservations, as outlined above.

# Response

**Published:** 2022-04-10

**Authors:** Gayani Amarasinghe

**Affiliations:** Rajarata University, Sri Lanka

We would like to express our gratitude to the reviewer for reviewing the manuscript. Please find a point-by-point reference to the comments that have been provided.

**Comment 1:**

 This study is the largest to date to be conducted in a single region of Sri Lanka to determine the associations of socioeconomic status, dietary history, and maternal anthropometric findings with the presence of anemia that was confirmed by blood testing.

 Anemia is confirmed by appropriate laboratory blood analysis. Survey data and nutritional information will be utilized. The published research will likely be helpful in creating information that can help inform public health policies that address proposed causation in Sri Lanka. Association of ≥ 10% will be reported.

**Reponse:**

 Thank you for the comment.

**Comment 2 :**

 Diagnosing anemia during the first trimester of pregnancy is a strength of the study, as anemia in the first trimester of pregnancy is associated with the poorest maternal and perinatal outcomes. The standard use of 120 mg of oral iron in Sri Lanka when iron deficiency is diagnosed is not consistent with recent data suggesting the negative impact of hepcidin on iron absorption.

**Response:**

 Thank you for pointing this out. We certainly agree that 120 mg of oral iron is not backed by the latest evidence. However, this was the practice according to the national maternal care guideline of the country at the time of proposal preparation and carrying out the study, which was not under the control of the researchers. We have clearly stated this in our manuscript as the current recommendation of the maternal care package as noted below:

 “anaemia screening during preconception and antenatal clinics (at their initial appointment and 28 weeks), universal iron supplementation, provision of double dose iron (elemental iron 120 mg/day) for anaemic mothers, nutritional education, prophylaxis for malaria, and treatment for worm infestations **have long been incorporated to Sri Lanka’s pregnancy care package**”.

**Comment 3:**

 The study is not generalizable, being too costly to implement on a population basis.

**Response:**

 Thank you for this comment. We agree that the process would be costly if it were to be used for routine surveillance. We do not propose to generalise the study methodology or etiological investigations to the entire population. Rather, this is an epidemiological study where we want to find out what aetiologies cause anaemia in this community so that they can be addressed through different public health measures which may or may not include an etiological diagnosis.

**Comment 4:**

 Most studies, in contrast to the data presented, suggest that iron deficiency anaemia is by far the most common cause of anemia in pregnancy.

**Response:**

 Thank you. We agree that iron deficiency is the commonest cause of anaemia in many places. However, a significant percentage of anaemia is due to other aetiologies. We have not claimed other aetiologies will be the commonest cause.

 “This raises the question of whether etiologies other than iron deficiency are **significantly responsible for anaemia** in this population. Previous reports on the presence of hemoglobinopathies, heterogeneous nutrient deficiencies and hereditary disorders among Sri Lankans support this assumption ^24– 26^. A recent national-wide study also indicates that iron deficiency may contribute only to 34% of anaemia in pregnancy ^20^.”

**Comment 5:**

 Additionally, no mention of the use of intravenous iron is discussed as this therapy is becoming more widely used to address moderate to severe anemia during pregnancy.

**Response:**

 Thank you for highlighting this new trend. However, we do not think that mentioning it is very much relevant to the current study, which does not focus on treatment modalities for anaemia or iron deficiency.

**Comment 6:**

 It would be important to comment on the grade of anaemia (mild, moderate, severe) in the findings.

**Response:**

 Thank you for pointing this out. We have added the grading of anaemia as moderate, mild, and severe to the methodology section of the revised manuscript as you have suggested:

 “Anaemia will be further classified as mild (10 – 10.9 g/dl) moderate (7-9.9 g/dl) and severe (<7 g/dl) based on the WHO classification.”

**Comment 7:**

 Is the rationale for, and objectives of, the study clearly described? No

**Response:**

 To clarify the objectives of the study we have modified the final paragraph of the introduction section as follows:

 “This study will explore aims to estimate the haemoglobin distribution and, the prevalence of anaemia, and identify factors associated with anaemia among first-trimester pregnant women in Anuradhapura district, Sri Lanka. It will also explore investigate the contribution of several etiological causes to anaemia in this population.”

**Comment 8:**

 Are the datasets clearly presented in a useable and accessible format? No

**Response:**

 This is the protocol paper and we do not present any data here.

# Peer review: Reviewer response for version 2

**DOI:** 10.5256/f1000research.56421.r85340

**Published:** 2021-06-17

**Authors:** Manjula Danansuriya

**Affiliations:** 1 Anti Malaria Campaign, Ministry of Health, Colombo, Sri Lanka

After considering the revised text and the author's responses to the issues highlighted, I feel the article is in good shape now.

Is the study design appropriate for the research question?

Yes

Is the rationale for, and objectives of, the study clearly described?

Partly

Are sufficient details of the methods provided to allow replication by others?

Yes

Are the datasets clearly presented in a useable and accessible format?

Not applicable

Reviewer Expertise:

Community Medicine

I confirm that I have read this submission and believe that I have an appropriate level of expertise to confirm that it is of an acceptable scientific standard.

# Peer review: Reviewer response for version 1

**DOI:** 10.5256/f1000research.31221.r81701

**Published:** 2021-04-15

**Authors:** Manjula Danansuriya

**Affiliations:** 1 Anti Malaria Campaign, Ministry of Health, Colombo, Sri Lanka

1. Abstract body should be homogenous. Objectives are stated differently at different places. Propose to improve the title as -assessing/exploring prevalence of known causes of anaemia- rather than "assessing etiology".
2. Justification could have been backed by more stronger reference from 2015 National nutrition survey which revealed - prevalence of anaemia among preg. women as 31.8% ; of them only 9.9% were identified as having iron def. anaemia (IDA) as per serum ferritin levels, highlighting other causes for anaemia is more prevalent among SRL pregnant women compared to IDA.
3. Sample size should be 865 not 383. Equation should be:
4. Ideally sample size should have been calculated to different entities of anaemia (as per available literature) i.e.: thalassemia, mixed deficiency, etc and sample size for the study should be the largest N of those ( see here).

Is the study design appropriate for the research question?

Yes

Is the rationale for, and objectives of, the study clearly described?

Partly

Are sufficient details of the methods provided to allow replication by others?

Yes

Are the datasets clearly presented in a useable and accessible format?

Not applicable

Reviewer Expertise:

Community Medicine

I confirm that I have read this submission and believe that I have an appropriate level of expertise to confirm that it is of an acceptable scientific standard, however I have significant reservations, as outlined above.

# Response

**Published:** 2021-04-23

**Authors:** Gayani Amarasinghe

**Affiliations:** Rajarata University, Sri Lanka

We would like to express our gratitude for the valuable  comments given by the reviewer. Please find a point by point reference for the comments below.

**Comment 1:** Abstract body should be homogeneous. Objectives are stated differently at different places. Propose to improve the title as -assessing/exploring prevalence of known causes of anemia- rather than "assessing etiology".

**Response:**  Thank you for pointing out the discrepancy in stated objectives at different places.  We have corrected the objectives in the abstract and the main text to improve the clarity and homogeneity. We would like to adhere to the original title of the article rather than updating to ‘exploring prevalence of known etiologies’ as our original aim is to actually identify etiologies that may matter in the study population. That’s why the algorithm includes examination of peripheral blood film assessments (in hope of identifying clues to alternate etiologies) when the suspected etiology proves unlikely according to the algorithm based testing. 

**Comment 2:** Justification could have been backed by stronger reference from 2015 National nutrition survey which revealed - prevalence of anaemia among preg. women as 31.8% ; of them only 9.9% were identified as having iron def. anaemia (IDA) as per serum ferritin levels, highlighting other causes for anaemia is more prevalent among SRL pregnant women compared to IDA.

**Response:  **Thank you for this valuable suggestion. We updated the text accordingly.

**Comment 3:** Sample size should be 865 not 383. 

**Response:** Thank you very much for pointing this out. We have used the same formula for calculation. However, we have mentioned that we used 2% precision for the calculation whereas we had actually used 3% precision. This has been a mistake from us and has been corrected in the new version. 

**Comment 4:** Ideally sample size should have been calculated to different entities of anaemia (as per available literature) i.e.: thalassemia, mixed deficiency, etc. and sample size for the study should be the largest N of those (see here).

**Response:** We agree that this would have been the ideal method. However, when the known population prevalence of etiologies (iron deficiency and thalassemia) among pregnant women are applied for the sample size calculation, obtained minimum sample sizes are smaller than the sample size we have used (n-1866). Hence we employed this method also taking into consideration the feasibility.
